# Enhanced Changeover Detection in Industry 4.0 Environments with Machine Learning

**DOI:** 10.3390/s21175896

**Published:** 2021-09-01

**Authors:** Eddi Miller, Vladyslav Borysenko, Moritz Heusinger, Niklas Niedner, Bastian Engelmann, Jan Schmitt

**Affiliations:** Institute Digital Engineering (IDEE), University of Applied Sciences, Würzburg-Schweinfurt, Ignaz-Schön-Strasse 11, 97421 Schweinfurt, Germany; eddi.miller@fhws.de (E.M.); vladyslav.borysenko@fhws.de (V.B.); moritz.heusinger@fhws.de (M.H.); niklas.niedner@fhws.de (N.N.); jan.schmitt@fhws.de (J.S.)

**Keywords:** machine learning, changeover, human–machine interaction

## Abstract

Changeover times are an important element when evaluating the Overall Equipment Effectiveness (OEE) of a production machine. The article presents a machine learning (ML) approach that is based on an external sensor setup to automatically detect changeovers in a shopfloor environment. The door statuses, coolant flow, power consumption, and operator indoor GPS data of a milling machine were used in the ML approach. As ML methods, Decision Trees, Support Vector Machines, (Balanced) Random Forest algorithms, and Neural Networks were chosen, and their performance was compared. The best results were achieved with the Random Forest ML model (97% F1 score, 99.72% AUC score). It was also carried out that model performance is optimal when only a binary classification of a changeover phase and a production phase is considered and less subphases of the changeover process are applied.

## 1. Introduction

In a study by the Leibniz Centre for European Economic Research (ZEW), it was carried out that digitalization in SMEs in Germany is progressing slowly (SME: small and medium-sized enterprise [1] (p. 4), [2]. With the intention to support and digitalize the system landscape of these metalworking companies, the OBerA project (OBerA: “Optimization of processes and machine tools through provision, analysis, and target/actual comparison of production data”) was founded. The consortium is formed by five production-oriented companies from the region of Franconia in northern Bavaria, Siemens as a technology partner, and the University of Applied Sciences Würzburg-Schweinfurt as an academic partner (for a detailed description of the consortium, please see [3]).

One focus of the research project is to improve the availability management on the shopfloor. Especially, the changeover times of machines were identified as a major working task in terms of availability management. A use case was built up at the Pabst GmbH company from the consortium, which manufactures mechanical components by drilling, milling, and grinding processes in small lot sizes. As a consequence, the machine tools need to run changeover procedures several times a day. The transparency of the changeover process to facilitate availability management so far is limited. These changeover processes are mostly done manually, and the changeover interval is not documented. Additionally, different workers are involved in this procedure during different shifts. 

Therefore, the research technique is to increase the transparency of changeover processes to support the availability management on the shopfloor. This shall be achieved by Machine Learning approaches, which are applied to identify changeover processes out of a big dataset generated by external sensors, which are attached to real production machines. In Section 2 of this article, the former research work of the authors in the field of changeover detection with machine learning is summarized. Section 3 explains how the preliminary research work was enhanced to further improve the Machine Learning model performance. In Section 4, the implementation of the enhancements is described. At last, in Section 5, the results of the enhancements are discussed. Section 5 also summarizes the article and provides an outlook to further research activities.

## 2. Summary of Preceding Research Work

In a former article, it was described that changeover times are one of the main reasons to decrease the Overall Equipment Effectiveness (OEE) of production facilities. To improve transparency in the changeover processes, a machine learning (ML) approach was presented to distinguish between changeover and production phases by a setup of external sensors, which can be applied to existing antiquated machines. The corresponding sensor setup was established at a milling machine (DMG 100 U duoBLOCK) of the company Pabst GmbH from the OBerA project consortium. The company Pabst GmbH belongs to the group of small to medium-size companies (SME). Multiple machine learning models such as Ensemble Classifiers, Support Vector Machines (SVM), Naïve Bayes, Logistic Regression, and Decision Tree algorithms were applied. A “Fine tree” model as a Decision Tree algorithm performed with a value of 0.92 for the performance indicator “Area Under the Curve” (AUC, not suited: 0; ideal: 1), but the true/false positive rates of the “Changeover” class were both around 50%, meaning that a “Changeover” event was often erroneously predicted as “Production” (48.3%), and on the other hand, a true “Changeover” event was only predicted correctly with 51.7%. In comparison, a RUSBoosted tree as an Ensemble Classifier achieved much better true/false positive rates for the “Changeover” class (false positive rate: 11.6%, true positive rate: 88.4%) with an AUC score of 0.93. In conclusion, it was pointed out that a detection of changeover phases with a heterogeneous sensor setup is feasible, and as a further research goal, the refinement of the classification approach was proposed. On one hand, this implied improvement of the boundary conditions for the machine learning model i.e., the sensor setup, and on the other hand, it indicated the need to make use of more detailed subphases for the general “Changeover” phases [3].

## 3. Setup and Enhancement of the Research Technique

To add more flexibility and transparency, the entire programming project was shifted from the MATLAB “Classification Learner” environment to Python Jupyter notebooks. It was possible to reproduce the results from [3] in a Python environment. All but one of the algorithms were successfully replicated in Python using the scikit-learn library, while the RUSBoosted Decision Tree was built using the imbalanced-learn library, which is fully compatible with scikit-learn API. The MATLAB “Classification Learner” operates via presets and then automatically finds optimal parameters. When transferred to Python, the same parameters were manually assigned to similar algorithms in an attempt to copy MATLAB presets to get the same results in the Python environment before any improvements or adjustments were made (see Table 1).

MATLAB displays the performance of each model via accuracy. Therefore, the same metric was used when evaluating models in Python. It is important to note that the parameter accuracy is not the best metric for the used test dataset and was only used to compare models within two environments and not their overall performance. For more details on ML performance metrics, see Section 5.2. Table 2 shows the obtained results from MATLAB and Python with the same test dataset. In all cases but the Linear SVM model, the results from MATLAB were replicated and even improved. However, the Linear SVM shows only a 0.1% worse performance in Python than in MATLAB:

In the former research, the research task was to distinguish between “Changeover” and “Production” with a binary classification in a supervised learning approach [3]. The supervision is conducted by a researcher who oversees the complete changeover and production process and records timestamps for the specific phases. Then, these timestamps are assigned to the sensor data, which were recorded during the supervision (“labeling”). Then, the machine learning models were trained with this labeled data (“supervised learning”). From a production planning point of view, this approach makes it possible to derive starting and stop times of the changeover by applying the trained machine learning model later with current production sensor data. These can be used to improve production planning or adjust product cost calculation. Hence, a binary classification between “Changeover” and “Production” is sufficient for this requirement. From the point of view of manufacturing engineering, more subphases for changeover could create more knowledge of the entire changeover process to facilitate further optimizations of changeover times. Accordingly, the supervisor has more efforts in labeling more than two phases, and the machine learning algorithms must conduct a multiple classification. After weighing the pros and cons, it was decided to include more than two changeover phases in the supervision and labeling process (see Section 3.1).

Tran et al. discuss in [4] the potential of Indoor Positioning Systems (indoor GPS) and its applicability to Lean Management concepts. They identify the high potential of indoor GPS for setup and changeover. As the changeover process at the company Pabst is conducted manually, it was decided to also integrate an indoor GPS into the sensor setup to track the position of the worker while conducting the changeover and production process (see Section 3.2).

Section 3.3 describes the data-handling process, while Section 3.4 explains the data preparation. 

**Figure 1:**
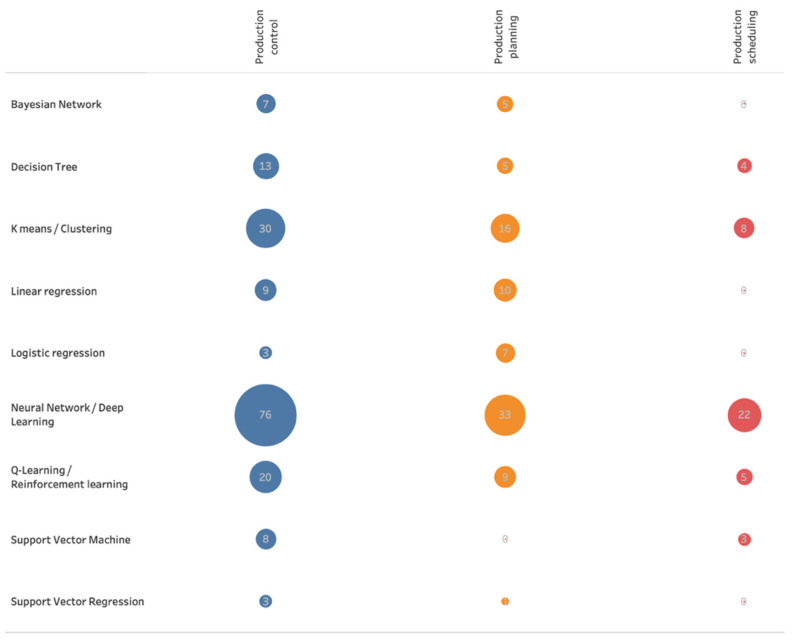
Search results by machine learning algorithm and application area (adapted from [5]).

Vojdani and Erichsen carried out a literature review to determine the potential of different machine algorithms in different production-related applications areas. As application areas, “Line feeding/line stocking/kitting/part feeding”, “production planning”, “production control”, “inventory/warehouse”, “production scheduling and sequencing”, and “transport” were identified. The following Figure 1 shows the number of search results of machine learning algorithms in the areas “production planning”, “production control”, and “production scheduling and sequencing” [5]:

Among all algorithms, the application of Neural Networks/Deep Learning algorithms was most frequently counted in the search results of this literature research. However, in the former research work, the best classification results were achieved with a Decision Tree algorithm and an Ensemble Classifier. It was also decided to include Neural Networks in the further research technique (see Section 4).

### 3.1. Changeover Phases

According to VDI guideline 3423 [6], the occupied time T_B_ of a single machine consists of the following:

- Utilization time T_N_;
- Test time T_C_;
- Preventive Maintenance time T_W_; and
- Organizational and Technical Downtime T_O_ and T_T_.

The time for changeover is part of the Organizational Downtime T_O_, which consists of the following:

- Downtime from short-term lack of jobs T_A_, and
- Time needed for setup of the machine T_R_.

Hence, the time for changeover is covered by the parameter T_R_. According to the definitions above, the time period of changeover can be considered as T_R_, and the remaining time consists of all the other parameters mentioned above. In this case, two phases need to be classified by an ML algorithm. Depending on the underlying time model, there are more phases thinkable: e.g., in the ERP system SAP, additionally, a teardown time after the processing and a setup time during processing are considered.

With the consortium of the OBerA project, a workshop was carried out to find subphases of their individual changeover processes. These phases were organized into three groups, including the starting phase of changeover, the main phase, and the end of the changeover process. Table 3 shows the collected 19 subphases in the workshop. For each subphase, the partner of the consortium who conducts the specific subphase is added in brackets, e.g., “BFKP”, meaning that the companies Brehm, Franken, Kritzner, and Pabst conduct this subphase. The subphases were assigned to the three groups having preparation activities mostly covered by the starting phase, the running and optimization of the NC program in the main phase, and manual component treatment and closing actions in the ending phase. The subphases **7**+8, **14**+15, and **16**+17 were grouped together to facilitate the later labeling process with longer phase durations.

For the machine learning task, it was decided to classify three different phase concepts:

- Two-phase approach (No. 1: Changeover including intermittent idle time, No. 2: Production phase);
- Five-phase approach (No. 1: Starting phase, No. 2: Main phase, No. 3: Ending phase, No. 4: Idle/break phase, No. 5: Production phase); and
- Twenty-one-phase approach (No. 1–19 Subphases from Table 3, No. 20 Idle/break phase, No. 21 Production phase)

The machine learning approach, which is applied in this article, belongs to the supervised learning. In this case, a supervisor tracks the changeover processes while the data of the connected sensors are recorded. During the tracking, the supervisor selects one of the 21 subphases from the list, which covers all the current changeover situations and assigns at same time the current timestamp. With these timestamps, later on, the recorded sensor data can be labeled with the subphases that occurred in the changeover process. Then, these labeled data are used to train a machine learning model.

Then, the five-phase approach is labeled by assigning a Starting phase, Main phase, and Ending phase according to Table 3. Accordingly, the two-phase approach can be finally labeled as a holistic changeover phase consisting of a Starting phase, Main phase, and Ending phase. 

### 3.2. Adjusted Sensor Concept

The initial work of changeover detection started with the measurements taken by the following five sensors, which were mounted onto one of the milling machines of the company Pabst [3]. The machine is a machining center and allows for a complete processing of parts with complex geometries. It is equipped with an automatic tool changer and can perform tasks such as turning, milling, drilling, and grinding.

**Figure 2:**
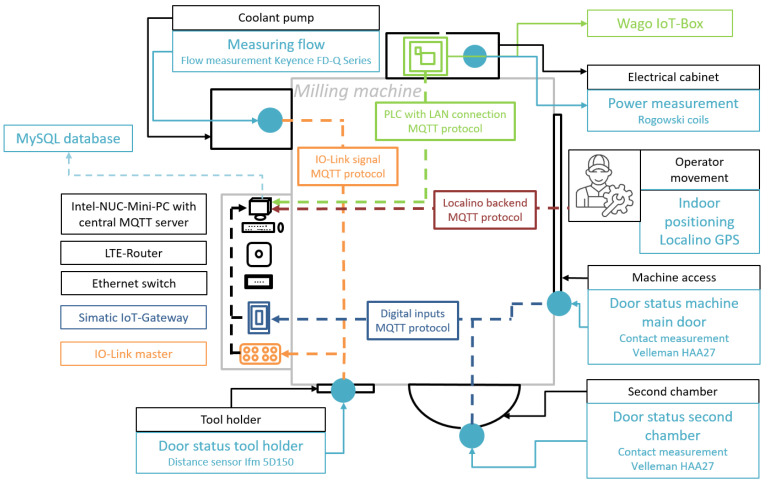
Milling machine and sensor setup with added GPS monitoring.

To check the status of the tool holder door, an Ifm 5D150 distance sensor was installed. It measures the distance from its mounting point to the handle of the tool holder door. When the operator opens the door, the measured distance increases, and the sensor recognizes the door as being opened. As the door closes, the distance decreases, the operator leaves the tool holder, and the tools have been changed. To further monitor the machine activities, the coolant flow was chosen. The Keyence FD-Q Series measures the flow of coolant liquid via IO-Link interface. The sensor is mounted directly on the coolant pipe. As soon as the machine starts working and pumping coolant, the sensor detects the flow. The next vital points of the machine are the workpiece chambers. The milling machine is equipped with two separate chambers, which both have to be observed. In order to register when the operator opens one of the two doors, two pairs of Velleman HAA27 contact switches were placed. Each pair consists of two magnet switches that generate a signal when coming close to each other. Finally, to measure the power output of the milling machine, the Wago IoT-Box 9466 was considered. This IoT-Box is a ready-to-use three-phase power measurement module that uses Rogowski coils. The coils are mounted directly onto the three power phases of the machine in the electrical cabinet. Then, it enables observing the machine data such as the strength of the electric current, voltage, and power. The sensor concept is illustrated in Figure 2.

Even when using all the described sensors above, there are still changeover steps that cannot be easily monitored or recognized. In particular, subphases 13 and 14 of the Ending phase of Table 3 are difficult to cover by the sensors described so far, which are attached to the machine. As the mentioned activities are conducted at workplaces nearby, additional GPS location data were utilized to cover these phases. To monitor the position and behavior of the machine operator, a Localino indoor tracking system was used. The Localino system consists of multiple anchors and a tag. The anchors are placed around the milling machine to triangulate the signal of the tag. At the beginning, the anchors were spread further inside the production hall to increase the covered area, but the massive milling machine was blocking the signal. As a result of this, the positions of the anchors were limited to a smaller area around the machine where the operator was working. After placing and activating the anchors, the tag can also be activated. The tag is immediately recognized by the anchors and writes the coordinates of the operator who is holding the tag into the Localino service backend that is running on the NUC mini PC. Table 4 shows all used sensor systems with the corresponding measuring object and measuring type.

### 3.3. Data Handling Concept

To provide internet access to all sensors via LAN, an LTE-Router in combination with an Ethernet switch is used. Both the Ifm 5D150 and Keyence FD-Q Series are IO-Link devices, which need to be connected to an IO-Link master. It controls the communication of all plugged-in IO-Link devices. The Velleman HAA27 contact switches on the other hand cannot be connected via IO-Link. Therefore, a Simatic IoT2040 gateway was installed. The contact switches are directly attached to the gateway, which then reads the digital signals generated by the contact switches. The Wago IoT-Box, as a ready-to-use application, has a built-in PLC and has access to different protocols. The Localino setup creates its own Wi-Fi network to exchange data between beacons and tags and then is connected to the LTE router, which forwards the collected data to the backend. The backend is a service, which is running on an NUC mini PC. The whole setup revolves around using the MQTT protocol. Each sensor is capable of transmitting data via MQTT. The NUC mini PC is equipped with a central MQTT server and Node-Red, which provides a graphical programming interface to integrate IoT equipment. It allows for a convenient way to subscribe to the different data message topics and to transform the acquired sensor data into a usable format. At last, after being received and processed by Node-Red, the data are sent and stored into an SQL database. The rows and columns of the data are configured to display the ID, timestamp, topic, value, unit, and sensor name. Furthermore, for the purpose of ML classification, each dataset is labeled with the phase label according to the corresponding phase approach (see Section 3.1).

### 3.4. Adjusted Data Preparation

The measured values are transmitted from the described experimental setup directly into the SQL database. This means that every second, one row for each sensor value is written into the database. However, to process the data in a machine learning model, the data structure has to be changed, since the raw data from SQL provide readings per second in seven separate rows instead of just one with all the sensor data. Therefore, a Python script that retrieves sensor data from MySQL database and creates a unique table for each sensor was coded. Afterwards, all the tables were combined into one using the outer join on the timestamp. This way, a table with the timestamps as well as the different sensor values as columns was created. While joining all tables, all sensor columns were also renamed to human-readable names.

Initially, the new data frame showed missing values especially in the columns of the Localino sensor, be it due to sensor data acquisition frequency or as a result of outer join. Simply deleting the corresponding rows of the table resulted in an enormous loss of training data. For this reason, the gaps were filled with the moving average of 10 measured values. For the sensors of power, coolant, and the status of the machine doors, rare gaps occurred in the range of 1–2 s (equivalent to two timestamp rows), and they were filled with the preceding last valid value from the specific column.

In the next step of data preparation, the measured values of the Ifm 5D150 distance sensor were converted from the file format float to a binary signal to match the data characteristic of the other two door sensors. As a part of Z-Transform, the data were also clipped, meaning that all the values beyond thresholds of three standard deviations were rounded up/down to the value of the threshold. This way, outliers were eliminated, and the data were prepared to the next step of standardization using scikit-learn StandardScaler. StandardScaler standardizes features by removing the mean and scaling to unit variance, thus ensuring the data are normally distributed and have a mean of zero [7]. 

The resulting data frame contained a total of 39.591 rows. Then, this data frame was labeled with the data of a changeover workshop in the period under consideration using the setup phases described in Section 3.1. The final table was finally transferred to a new SQL database to make it permanently available.

## 4. Application of Machine Learning for Changeover Detection

As described in Section 2, in the former research work, the machine learning models Ensemble Classifiers, Support Vector Machines (SVM), Naïve Bayes, Logistic Regression, and Decision Tree algorithms were applied. For the further research, Decision Trees, Support Vector Machines, and (Balanced) Random Forest algorithms were chosen. In Section 3, it was explained that the frequent application of Neural Networks in production optimization-related publications led to the additional consideration of Neural Networks to the choice of machine learning models, which shall be covered by this research work.

In Section 4.1, the application of Neural Networks is described. In the subsequent sections, Decision Trees (Section 4.2), Support Vector Machines (Section 4.3), and (Balanced) Random Forest (Section 4.4) are explained.

### 4.1. Neural Networks

Artificial Neural Networks (ANN) are modeled in the way the human brain works. Neurons are the working units of the network. These units process input values in three steps. In the first step, the input values are weighted. Then, the resulting network input is processed in what is called the actuation function. In the last step, the network output is determined depending on a threshold value [8], (pp. 19–194).

ANNs consist of a large number of such neurons, which are structured in layers and interact with each other via directed and weighted connections. Neural Networks usually consist of an input layer, one or more hidden layers, and an output layer. The input layer processes the input values of the analysis. The output of the input layer neurons results in an activation of the hidden layer neurons. It is to be mentioned here that the activation degree of the hidden layer neurons is determined independently during the training process. The programmer only determines the hyperparameters of these layers, but he does not exert any influence in the form of predefined relations on the neurons of these layers. The output layer determines the target variable of the network [9] (p. 302).

The neuron weights are adjusted in the training process. This process is often performed with the backpropagation algorithm, which minimizes the total error of the network iteratively using the gradient method. ANN that work like this are called Recurrent Neural Networks (RNN) [10], (p. 712).

In this research work, an RNN with two hidden layers for classification is used. The model was developed with the Python library TensorFlow. In order to optimize the hyperparameters of the network, a grid search has been applied. The following Table 5 shows the selected parameters as well as the optimal combination of these for the neural network.

### 4.2. Decisision Trees

A Decision Tree is a highly interpretable classifier that assigns a class to a data point based on the series of questions (conditions) posed about the features belonging to the data item. Decision Trees are also able to work with nonlinear data [11] (p. 5). 

**Figure 3:**
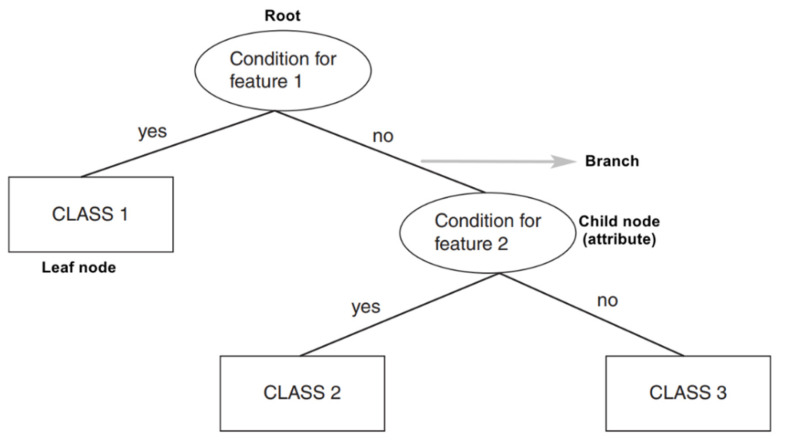
Generic Decision Tree (adapted from [11] (p. 5)).

Very much similar to a real tree, a Decision Tree has a root, branches, and leaves. A root node is the parent of all others, and it expresses the condition for the very first feature. At this node, the very first binary split happens, leading to the next questions. Each node represents a feature (attribute), each link creates a branch and leads to a decision (rule) and finally leads to an outcome (leaf node) [12] (p. 74). Figure 3 shows an example of a generic Decision Tree. The way nodes are defined and created is based on the node purity score. A 100% pure node is the one whose data belong to a single class, and a 100% impure one has its data split evenly between two classes. The impurity can be measured using entropy (classification), mean squared errors (regression), and Gini index [13] (p. 25). The idea behind this algorithm is to mimic the human way of thinking and decision making for a high interpretability [12] (p. 74). However, this comes with a price of Decision Trees being sensitive to changes in the features that can negatively affect their predictive performance [13] (p. 25). 

In the MATLAB implementation, these trees were called a fine tree, a medium tree, and a coarse tree (see Section 2). The main difference between them was the parameter responsible for the maximum number of splits, which in scikit-learn is called “max_leaf_nodes”. Replicating these Decision Trees in scikit-learn yielded the following results (see Table 6):

Based on this previous work, three Decision Trees were created for the two-phase, five-phase, and 21-phase approaches, respectively with scikit-learn’s DecisionTreeClassifier using standard hyperparameters [14]. The standard setting for “max_leaf_nodes” is “none”, which means an unlimited number of leaf nodes. Detailed results can be found in Table 8 in the later article.

### 4.3. Support Vector Machine

The Support Vector Machine (SVM) is a maximum-margin classification algorithm, which is able to separate nonlinear data by learning a hyperplane, which separates the data. If the data are nonlinear, the kernel trick is used to calculate similarities in a higher-dimensional space, which aims to make the data linear and separable. The goal is to learn support vectors that maximize the space between the two classes. The SVM is a binary classifier, which can be adapted to multiclass problems by using the one-vs.-the-rest approach [15] (pp. 326–339).

For the usage in the setup, the C parameter for the binary classification task was tuned. The parameter controls the strength of the regularization, which is inverse proportional to C, which resulted in C = 10,000 using the scikit-learn implementation [7].

For the implementation in Python, scikit-learn’s Support Vector Classification (SVC) was used [16].

### 4.4. (Balanced) Random Forest

The Random Forest (RF) is an algorithm from the field of supervised learning and can be used for classification and regression. Unlike the Decision Tree algorithm, the RF trains an entire ensemble of trees instead of one [17] (p. 157 f.). This approach has the advantage that the model is less susceptible to changes in the dataset [18] (p. 467), and overfitting is avoided [19] (p. 124). 

During the training process, a separate prediction is made for each tree in the forest. Then, the different results of the classifications are evaluated by majority vote. In this way, wrong predictions are omitted and correct ones are summed up. This results in an improvement of the model [19] (p. 123).

To achieve the highest possible accuracy of the RF, the correlations among the trees of the ensemble must be minimized. This can be achieved with the so-called Bootstrap Aggregation or Bagging [20] (p. 10 f.). The Random Forest is created using the Python library of scikit-learn, which provides the “RandomForestClassifier” package.

As in the case of the neural network, the hyperparameters are optimized with the grid search. Table 7 shows the tested parameters and the best possible combination of them:

Additionally, Balanced RF [21] was utilized, which is a version of RF using a bootstrapping technique to improve the performance on imbalanced data. At each iteration, the algorithm draws a bootstrap sample from the minority class and randomly draws the same number of cases from the majority class. Thus, the number of processed cases is equally distributed. In the experiments, the Balanced RF was configured to use 100 trees. The following table shows the tested parameters and the best possible combination of them.

## 5. Discussion

In this section, an in-depth look into the evaluation of independent variables and the techniques that were applied is provided. Furthermore, the comparison between the different ML algorithms in regard to the performance will be emphasized.

### 5.1. Evaluation of the Independent Variables

In this section, details on the independent variables are provided. For this purpose, the features in a two-dimensional space by using projection as well as embedding techniques are visualized. Furthermore, the correlation matrix is presented to show how the features influence each decision of the model. 

**Figure 4:**
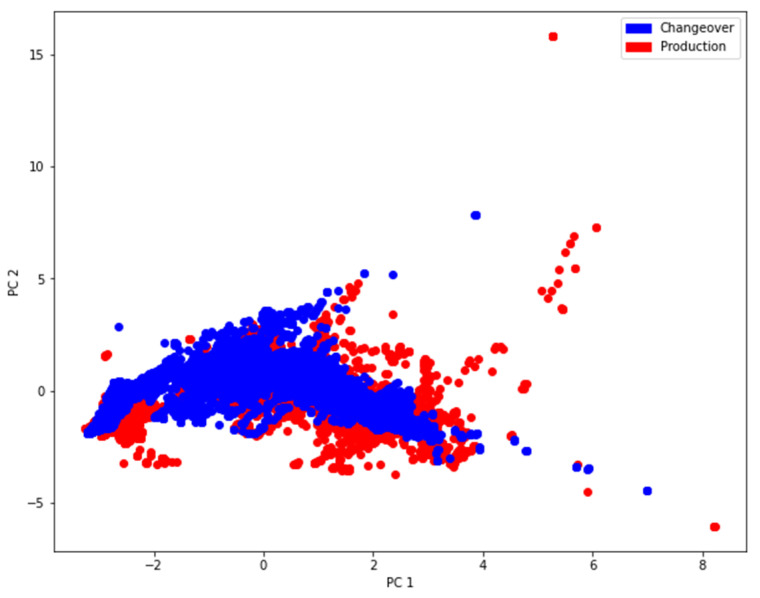
PCA showing the data projected on the eigenvectors corresponding to the two largest eigenvalues.

Figure 4 shows the data projected into a two-dimensional space by performing a Principal Component Analysis (PCA) on the preprocessed data. All columns are normalized to a mean of 0 and a standard deviation of 1 to work well with the PCA [22]. However, it can be seen that the PCA did not separate the data according to their class labels. This led to the assumption that the data may not be linearly separable. Thus, in the following step, t-Distributed Stochastic Neighbor Embedding (t-SNE) is applied to perform a nonlinear neighbor embedding into two dimensions.

**Figure 5:**
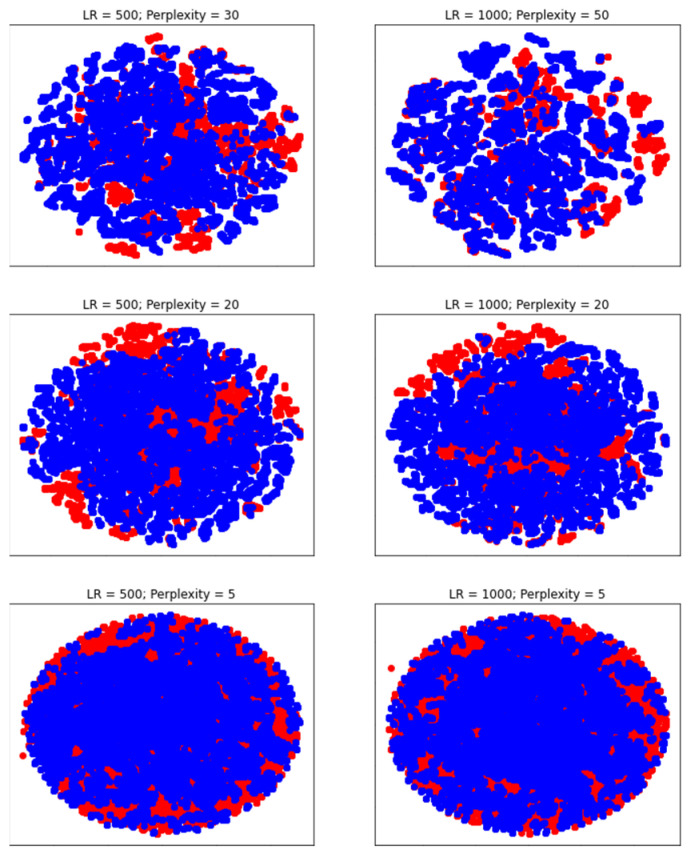
t-SNE plots of the data using different perplexity values and learning rates (LR). Red data points are representing the class “Production” and blue data points are representing the class “Changeover”.

Figure 5 visualizes the data using t-SNE with perplexity parameters between five and 50, as proposed in [23]; additionally, the learning rate of the algorithm was varied. The learning rate (LR) should be chosen between 10 and 1000 according to [23]. Different learning rates are applied in Figure 5. Additionally, learning rates between 10 and 500 were applied; however, this did not lead to visually better results. To speed up the computation of t-SNE on such a large dataset, the Barnes Hut method proposed in [24] instead of the original t-SNE [23] was used. As shown in Figure 5, it was not possible to distinguish the two classes clearly via t-SNE. Hence, it can be assumed that the data are not intrinsically low dimensional.

**Figure 6:**
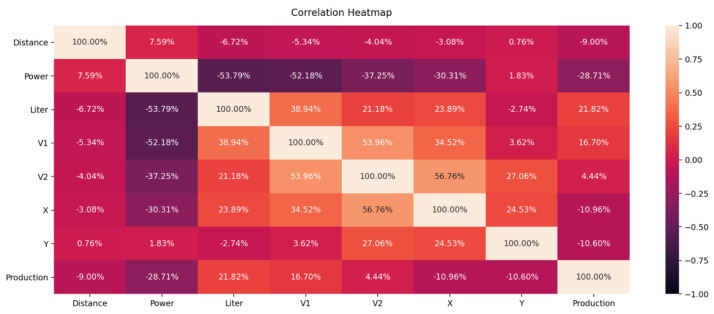
Correlation matrix of currently used in OBerA features (0.00: no correlation, ±1.00: high correlation).

Classical dendrograms as visualization techniques also provided no further insights, having around ten thousand rows of data, leading to a big and tangled dendrogram tree. Instead, a simple confusion matrix was created to analyze how and which features are correlated. As it can be seen from Figure 6, all the sensors have a considerable contribution to the decision-making process of the machine learning models.

All features show correlation coefficients in the range [−53.79%; 56.76%] which is indicating an average correlation. The distance door sensor (door status tool holder, see Figure 2) shows only one changing state over the complete changeover period, which results in a very low correlation in the range of [−9.00%; 7.59%]. 

In summary, all the described analyses show that the data belong to a higher dimensional problem and cannot be reduced on a subset of the independent variables.

### 5.2. Performance Metrics

The methods used to evaluate how well algorithms for classification are performing are divided into two categories: graphical and numerical. Graphical methods are plots that are interpretable by humans, while numerical methods present a single number to describe the performance of a classifier [25] (p. 462).

The most commonly used evaluator in machine learning nowadays is accuracy, as it shows the overall performance of an algorithm by presenting the probability of the true value of the class label [26] (p. 1018). The closer accuracy is to a value of 1 (or 100%), the better the algorithm is performing in general.

However, for imbalanced datasets, accuracy proved to be a rather misleading performance indicator due to the fact that the minority of classes have less weight than those of the majority. This negatively affects the classifier when performing on rare classes. Skewed data lead to skewed results [27] (p. 8).

Two other known metrics are precision and recall (sensitivity). Precision evaluates how good the predictive power of the algorithm is, while recall gives information on how effective the algorithm is on a single class [26] (p. 1018). Precision tells how correct the classifier was when classifying samples as positives, while recall shows how well the samples that needed to be classified as positive were classified as such [25] (p. 467). 

Neither precision nor recall are better metrics for an imbalanced dataset than accuracy due to their specific focus on the respective aspects. However, there is another metric called the F1 score, which is a weighted average of precision and recall. Both precision and recall have an equal relative contribution to the F1 score. Regarding accuracy, the F1 score scales from 0 to 1 (100%) [28].

In the upcoming performance comparison of the algorithms, the AUC indicator is also applied (AUC = 0: not suited; AUC = 1: ideal: 1). It was used in the previous research work and was introduced in Section 2. Furthermore, the confusion matrix technique, which shows the classification performances, is applied. Details for this technique can be found in [3]. 

### 5.3. Comparison of the Results

Table 8 compares the performance of the algorithms Neural Network, Decision Tree, SVM, Balanced Random Forest, and Random Forest on the classification of two phases, five phases, and 21 phases. It can be seen from Table 8 that binary classification (two phases) yields the best results. With additional phases, the performance of all algorithms decreases. Balanced RF shows a loss of 64% and Neural Networks show a loss of 35% from two-phase classification to 21 phases. Random Forest shows superior results for all phases classification, losing only 4% from two-phase to 21-phase classification. As an overall conclusion, it can be derived that Decision Trees and (Balanced) Random Forest yield the leading results.

**Figure 7:**
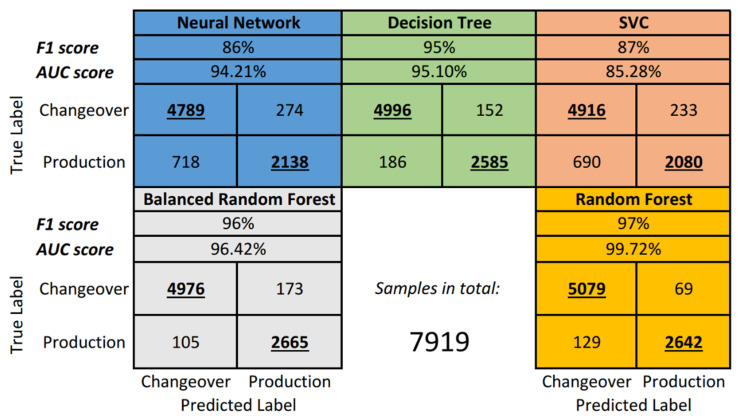
Detailed performance of each OBerA model.

For further comparison, Figure 7 presents the classical confusion matrices for all algorithms as well as the Area Under the Curve (AUC) performance indicator.

From the Figure 7, it can be seen that despite the relatively high AUC score (94.21%), the Neural Network performed with 86% F1 score. The confusion matrix provides a better overview by showing that the Neural Network misclassified 718 production samples as changeover. 

On the other hand, SVM/SVC performed better in terms of F1 score, but the AUC is 11% lower compared to the Neural Network and is actually the lowest among all models. The confusion matrix shows the same issue with production phase misclassification (690). 

The standard Decision Tree proved to be rather effective by achieving a 95% F1 score. In addition, there were fewer problems with misclassification despite the imbalanced dataset (186).

The Balanced Random Forest from the imblearn library shows even better results with slightly less production-phase misclassification (105) but slightly higher changeover-phase misclassification (173).

Lastly, the standard Random Forest ensemble algorithm has the best performance on the OBerA dataset, scoring 97%, and the lowest rate on changeover misclassification (129). A few more production labels are wrongly classified as changeover compared to Balanced Random Forest. However, the AUC score is very good with a value of 99.72%.

Finally, it can be concluded that the Decision Trees/(Balanced) Random Forest algorithms are performing very well on the underlying OBerA dataset. 

## 6. Summary, Conclusions and Further Research

After an introduction in Section 1, the preceding research work was summarized in Section 2: The ML model that was created in the former work showed a general feasibility for modeling changeover detection. Nevertheless, the model performance showed potential for improvements. In Section 3, all adjustments to the original approach were described. The complete ML project was transferred from MATLAB to Python to increase the flexibility in data processing. At the same time, the choice of ML algorithms was extended by Neural Networks. While in the former research work, only two phases were classified by the ML methods, the technique was broadened by five-phase and twenty-one-phase approaches (Section 3.1). Furthermore, the sensor setup was enhanced by an indoor GPS tracking system (Section 3.2). While applying the same data-handling concept as in the former research work (Section 3.3), the data preparation was adjusted (Section 3.4). To avoid a loss in training data, missing values were interpolated instead of dropping entire rows of data. In addition, the data for all the doors were converted into binary data (status open/close), outliers were eliminated, and scaling was applied. In Section 4, all the used ML methods were briefly explained, and the main hyperparameters for each ML method were introduced. At last, in Section 5, the research results were presented. In Section 5.1, it was shown that the set of chosen independent variables for the ML models cannot be reduced any further and represent the underlying problem completely. In Section 5.2, the chosen performance metrics for ML methods were explained and applied in Section 5.3. The Random Forest ML method showed the best performance of all applied ML models (97% for two phases, 93% for 21 phases). While many other ML methods showed weaker performances the greater the number of changeover phases classified, the Random Forest ML method only showed small performance reductions. Similarly, the Decision Tree showed a very good performance (95% 2 Phases, 88% 21 Phases).

For the operational application of ML for the changeover detection, it can be concluded from the results that only the two-phase approach guarantees a high quality of detection. To derive general strategies for a company’s changeover process, this might be sufficient, e.g., for product calculations, totals for changeover times and production times are used. To support detailed ergonomic improvement techniques, an automatic detection system for a maximum of phases e.g., the twenty-one-phase approach, might be better suited. It needs to be clarified in future research work if the present state of the results is sufficient for this task.

To further validate the results of the presented work, the detection system needs to be used on different machines with the same manufacturing technologies (milling). When a validation is achieved, the transfer of the shown technique to other manufacturing technologies such as turning can be considered.

In the performance analysis of the different ML methods, the F1 score was utilized for the underlying imbalanced classification problem. As the supporting metrics, AUC score was chosen in place of MSE and RMSE, which do not provide the trade-off information between class data and can lead to suboptimal solutions in cases where class weights are not properly determined [29] (p. 5). There are further improved methods to use with imbalanced datasets, which are discussed in the paper of Chicco and Jurman [30] (p. 10), as well as in Akosa’s work [31]. Namely, the Matthews correlation coefficient (MCC) shall be used in the future research for model evaluation in case of imbalanced data.

**Figure 8:**
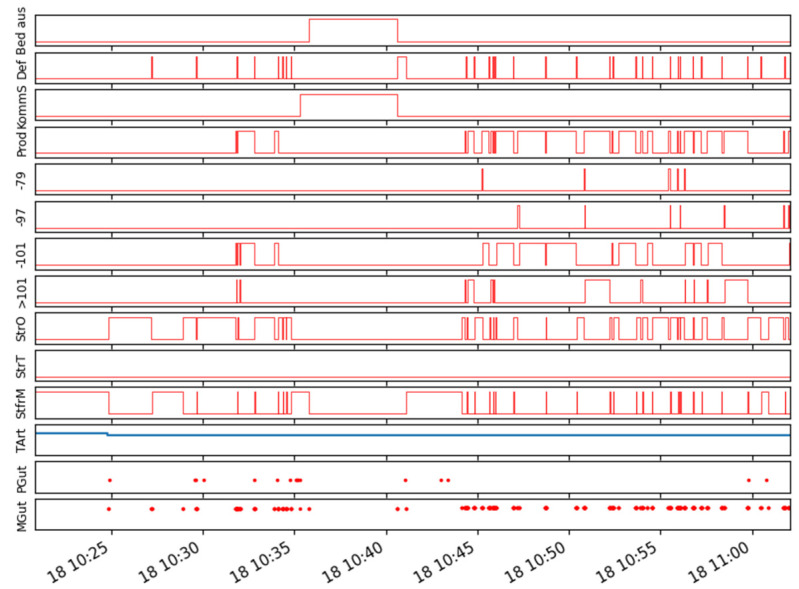
Signals from the internal machine control interface with SIEMENS AMP.

Finally, it needs to be pointed out that the shown technique is based on an external sensor setup. No data from machine controls were used so far. Neither were more sophisticated approaches such as AI or Human Action Recognition applied yet [32]. This approach was chosen as in SMEs, often old and heterogeneous machine landscapes are found, and machine controls often do not offer data interfaces. Figure 8 shows multiple signals from an UpToDate internal machine control data interface, which is established with the SIEMENS AMP interface (AMP: Analyze My Performance). In future research, the existing model shall be enriched by the usage of machine control data e.g., the configured override status, to further increase the performance of the changeover detection task.

## Figures and Tables

**Table 1:** Corresponding functions in Python to MATLAB Classification Learner.

MATLAB Classification Learner	Python Library	Python Function	Kernel/Settings
Fine Decision Tree	scikit-learn	DecisionTreeClassifier()	max_leaf_nodes = 100
Medium Decision Tree	scikit-learn	DecisionTreeClassifier()	max_leaf_nodes = 20
Coarse Decision Tree	scikit-learn	DecisionTreeClassifier()	max_leaf_nodes = 4
Logistic Regression	scikit-learn	LogisticRegression()	
Gaussian Naive Bayes	scikit-learn	GaussianNB()	Bernoulli’s had to be used
Kernel Naive Bayes	scikit-learn	BernoulliNB()	BernoulliNB()
Linear SVM	scikit-learn	LinearSVC(), SVC()	Linear
Quadratic SVM	scikit-learn	SVC()	Quadratic
Cubic SVM	scikit-learn	SVC()	Cubic
Fine Gaussian SVM	scikit-learn	SVC()	Radial Basis Function (RBF), gamma = 0.5
Medium Gaussian SVM	scikit-learn	SVC()	Radial Basis Function (RBF), gamma = 2
Coarse Gaussian SVM	scikit-learn	SVC()	Radial Basis Function (RBF), gamma = 8
Boosted Trees	scikit-learn	DecisionTreeClassifier()	AdaBoostClassifier()
Bagged Trees	scikit-learn	DecisionTreeClassifier()	BaggingClassifier()
RUSBoosted Trees	scikit-learn, imbalanced-learn	DecisionTreeClassifier()	RUSBoostClassifier()

**Table 2:** Accuracy comparison between MATLAB Classification Learner and Python.

Model	MATLAB Accuracy, %	Python Accuracy, %
Fine Decision Tree	92.8	93.4
Medium Decision Tree	91.6	92.8
Coarse Decision Tree	90.3	90.3
Logistic Regression	88.6	88.6
Naive Bayes	88.5	88.6
Linear SVM	88.7	88.6
Fine Gaussian SVM	90.8	93.8
Medium Gaussian SVM	89.0	95.4
Coarse Gaussian SVM	88.7	96.2
Boosted Trees	92.6	93.1
Bagged Trees	92.7	93.6
RUSBoosted Trees	79.5	80.6

**Table 3:** Derived subphases for changeover processes in the OBerA companies.

Starting Phase of Changeover	Main Phase of Changeover	Ending Phase of Changeover
(1) Logging on/off the setup job at the terminal (BFKP)	(11) Swiveling of machine tables (P)	(**14** * + 15) Cleaning of the component and dismantling of the component (BKP)
(2) Cleaning of the machine table (KP)	(12) Setting the zero point (BFKP)	(**16** * + 17) Deburring of the component and remeasurement of the component (BKP)
(3) Moving the fixture from machine table to workbench (KP)	(13) Running and optimizing the NC program (BFKP)	(18) Load the optimized NC program on the server (P)
(4) Fastening of component to fixture (BKP)		(19) Logging on/off the setup job at the terminal (BFKP)
(5) Transfer of fixture from workbench to machine table (KP)		
(6) Attachment of fixture to machine table (KP)		
(**7** * + 8) Loading the NC program (BFKP) and performing tool presetting (BKP)		
(9) Filling the tool magazine (BFKP) (10) Entering the current tool dimensions (BFKP)		

Companies: Brehm (B), Franken (F), Kritzner (K), Pabst (P); * only first number used for labeling.

**Table 4:** All sensors used in addition with GPS monitoring.

Sensor Type	Measuring Object	Measuring Type
Ifm 5D150	Door status tool holder	Distance measurement
Keyence FD-Q Series	Coolant flow	Flow measurement
Velleman HAA27	Door status machine main door	Contact measurement
Velleman HAA27	Door status second chamber	Contact measurement
Wago IoT-Box 9466	Machine power/performance	Power measurement
Localino indoor tracking	Operator GPS data	GPS measurement

**Table 5:** Parameters for recurrent neural networks.

Hyperparameter	Tested Variables	Best Result
Optimizer	SGD, Adagrad, Adadelta, Adam, Adamax, Nadam	Adam
Number of epochs	20, 30, 40, 50, 60	50
Batch size	15, 30, 50, 60, 100	50
Learning rate	0.001, 0.01, 0.1, 0.2, 0.3	0.001
Loss function	sparse_categorical_crossentropy	-
Number of neurons HL1	20, 30, 50, 100, 150, 200	150
Actuation function HL1	softmax, softplus, softsign, relu, tanh, sigmoid, hard_sigmoid, linear	relu
Number of neurons HL2	20, 30, 50, 100, 150	100
Actuation function HL2	softmax, softplus, softsign, relu, tanh, sigmoid, hard_sigmoid, linear	relu
Number of neurons output-layer	2	(for binary classification)
Activation function output-layer	softmax	softmax

**Table 6:** The settings of the Decision Trees in MATLAB and their performance in Python.

Tree Name	Max Number of Leaf Nodes	Python Accuracy
Fine tree	100	93.4
Medium tree	20	92.8
Coarse tree	4	90.3

**Table 7:** Parameters for Random Forest and Balanced Random Forest classification.

Hyperparameter	Tested Variables	Best Result
Max. depth of the trees	4, 10, 15, 30, 35	30
Bootstrap Aggregation (Balanced RF)	True, False	False
Number of Trees	40, 44, 48, 53, 57, 62, 66, 71, 75, 80	48
Number of features per split	‘auto’ = sqrt(n_features) ‘log2’ = log2(n_features)	auto
Min. number of data for internal node formation	2, 5	2
Min. number of samples at a leaf node	1, 2	1

**Table 8:** Performance of algorithms measured in terms of macro average F1 score.

Algorithm	F1 Score Two Phases	F1 Score Five Phases	F1 Score 21 Phases
Neural Network	86%	70%	51%
Decision Trees	95%	92%	88%
SVM	87%	81%	70%
Balanced RF	96%	70%	32%
Random Forest	97%	96%	93%

## Data Availability

The underlying dataset as well as the Python source code are available under https://github.com/ValdsteiN/OBerA-Enhanced-Changeover-Detection-in-Industry-4.0-environments-with-Machine-Learning.
